# Failure to Rescue and Lung Resections for Lung Cancer: Measuring Quality from the Operation Room to the Intensive Care Unit

**DOI:** 10.3390/cancers17172784

**Published:** 2025-08-26

**Authors:** Prokopis-Andreas Zotos, Vasiliki Androutsopoulou, Marco Scarci, Fabrizio Minervini, Ugo Cioffi, Andrew Xanthopoulos, Thanos Athanasiou, Dimitrios E. Magouliotis

**Affiliations:** 1 Department of Cardiothoracic Surgery, University of Thessaly, Biopolis, 41110 Larissa, Greece; zotospro@hotmail.com (P.-A.Z.); androutsopoulouvasiliki@uth.gr (V.A.); 2 Department of Cardiothoracic Surgery, Hammersmith Hospital, Imperial College Healthcare, National Health Service (NHS) Trust, London W12 0HS, UK; marco.scarci@nhs.net; 3 Luzern Kanton Hospital, 6000 Luzern, Switzerland; fabrizio.minervini@luks.ch; 4 Department of Surgery, University of Milan, 20122 Milan, Italy; ugo.cioffi@guest.unimi.it; 5 Department of Cardiology, University of Thessaly, Biopolis, 41222 Larissa, Greece; anxanthopoulos@uth.gr; 6 Department of Surgery and Cancer, Imperial College London, St Mary’s Hospital, London W2 1NY, UK; t.athanasiou@imperial.ac.uk; 7 Department of Cardiac Surgery Research, Lankenau Institute for Medical Research, Wynnewood, PA 19096, USA

**Keywords:** failure to rescue, FTR, thoracic surgery, lung cancer, lung resection, mortality, quality

## Abstract

Failure to rescue (FTR), defined as death following a potentially treatable postoperative complication, has emerged as a critical quality metric in thoracic surgery. In patients undergoing lung cancer resection, who are often at high risk due to comorbidities and limited pulmonary reserve, FTR significantly influences morbidity, mortality, recovery, and overall quality of life. This review explores the multifactorial nature of FTR in lung cancer surgery, highlighting key patient-related and system-level risk factors, such as surgical complexity, delayed complication recognition, inadequate escalation of care, and limited critical care resources. Existing models for patient rescue emphasize early detection and timely intervention, but often overlook the institutional and cultural changes required for sustainable improvement. Building on current evidence and integrating Kotter’s eight-step change model, we propose a novel multidimensional roadmap to reduce FTR through proactive monitoring, structured escalation protocols, multidisciplinary coordination, and continuous learning. Finally, reducing FTR in lung cancer resection requires more than clinical responsiveness. This necessitates a systemic transformation that aligns frontline practice with institutional readiness and a culture of safety.

## Simple Summary

Failure to rescue (FTR), defined as death after a potentially treatable postoperative complication, is increasingly recognized as a vital quality metric in thoracic surgery. In lung cancer resections, patients are particularly vulnerable due to comorbidities and reduced pulmonary reserve, making FTR a major determinant of morbidity, mortality, recovery, and long-term quality of life. This review examines the multifactorial drivers of FTR, including surgical complexity, delayed recognition of complications, insufficient escalation of care, and limited critical care resources. Current patient rescue models emphasize early detection and intervention but often fail to address broader institutional and cultural factors. To bridge this gap, we propose a multidimensional roadmap informed by Kotter’s eight-step change model, incorporating proactive monitoring, structured escalation pathways, multidisciplinary teamwork, and continuous learning. Reducing FTR requires more than clinical responsiveness; it demands systemic transformation that integrates frontline practice with institutional readiness and a strong culture of safety.

## 1. Introduction

Lung cancer continues to be the primary cause of cancer-related mortality in the United States, with approximately 130,000 deaths reported in 2022 alone [1,2]. Surgical resection remains the primary curative modality for patients with early-stage lung cancer; however, it carries a notable risk of postoperative complications. Recent literature indicates that overall complication rates following lung resection vary between 25% and 40%, with major complications observed in approximately 7% to 10% of cases [3,4,5,6,7,8]. Importantly, while these complications significantly impact patient prognosis, the rate of in-hospital mortality does not necessarily correlate with the frequency of these events. Rather, it is largely determined by the healthcare system’s ability to effectively identify and manage major complications, a concept referred to as failure to rescue (FTR).

In the context of lung cancer surgery, FTR describes the inability to successfully respond to and resolve serious postoperative complications, which can ultimately lead to outcomes such as respiratory failure or death. To mitigate this risk, it is imperative that institutions adopt targeted strategies to improve rescue efforts for patients experiencing adverse postoperative events. These strategies include establishing standardized protocols for early detection and response, fostering multidisciplinary communication, reinforcing technical and non-technical surgical training, and enhancing perioperative care pathways. Additionally, structured postoperative surveillance should emphasize prompt assessment of respiratory function and rapid intervention for critical complications such as pneumonia, pulmonary embolism, or acute respiratory insufficiency. Commitment to ongoing quality assessment and integration of evidence-based practices is vital for reducing FTR and improving overall outcomes in lung cancer surgery.

The concept of failure to rescue, defined as in-hospital mortality following the onset of a major postoperative complication, was initially introduced in the 1990s as a hospital quality metric [9]. Over the subsequent two decades, research consistently demonstrated that FTR rates were influenced more by institutional characteristics, such as hospital surgical volume, academic affiliation, nurse staffing ratios, and the availability of specialized care services, than by the complication rates themselves [10,11]. In more recent years, interest in FTR has expanded within the field of cardiothoracic surgery, where it is increasingly recognized as a meaningful indicator of postoperative care quality [11].

Beyond its clinical relevance, failure to rescue also resonates with broader organizational and system sciences. In oncology, frameworks emphasizing the psychosocial and institutional context of patient care have highlighted that outcomes are shaped not only by individual risk factors but also by systemic capacity for timely intervention [12]. Similarly, structured quality frameworks used across diverse medical fields reinforce the importance of standardized definitions and escalation protocols to minimize variability in adverse event management [13]. Insights from reliability engineering further suggest that complex healthcare systems, such as those delivering thoracic surgery, require continuous monitoring and feedback loops to reduce the probability of rescue failure [14]. Incorporating these perspectives enriches the conceptual basis of this review and underscores the need to link clinical outcomes with organizational change theory.

Despite this growing body of research, data specifically focusing on FTR in patients undergoing lung cancer resection remain limited. In particular, the influence of patient-level factors on the likelihood of successful rescue after postoperative complications has not been extensively explored in this population. The earliest application of FTR analysis in cardiac surgery was by Pasquali and colleagues [15], marking the beginning of a broader appreciation of its utility in evaluating surgical quality. Since then, FTR has gained momentum as a critical benchmark in the cardiovascular and thoracic surgical disciplines [10]. Although FTR has been increasingly recognized as a meaningful quality indicator in thoracic surgery, previous reviews have focused primarily on clinical determinants and outcome disparities. To date, no study has systematically examined FTR in lung cancer surgery through the lens of organizational change theory. By integrating Kotter’s eight-step model into the analysis, this review addresses a critical gap by translating clinical observations into structured, sustainable institutional strategies for reducing rescue failure.

This review aims to synthesize current evidence on failure to rescue following lung cancer surgery, with a dual objective: first, to identify patient-level, surgical, and system-level determinants of FTR; and second, to evaluate how organizational change frameworks, particularly Kotter’s eight-step model [16], may inform institutional strategies to improve rescue processes. To our knowledge, this is the first review to systematically integrate clinical determinants of FTR with change management theory in thoracic oncology.

## 2. Materials and Methods

### 2.1. Search and Articles Selection Strategy

This review was conducted following a predefined protocol agreed upon by all contributing authors and in adherence with the guidelines outlined in the Preferred Reporting Items for Systematic Reviews and Meta-Analyses (PRISMA) [17]. A comprehensive literature search was carried out using three major databases: PubMed (Medline), Web of Science (WoS), and Scopus (ELSEVIER), with the final search performed on 15 August 2025. The search strategy employed a broad combination of keywords, including: “lung cancer,” “lung resection,” “pulmonary resection,” “lobectomy,” “segmentectomy,” “NSCLC,” “failure to rescue,” “failure-to-rescue,” “FTR,” and “quality metric.”

Studies were eligible for inclusion if they met the following criteria: (1) original research articles involving more than 10 patients; (2) publication dates between 1992 and 2024; (3) English language; (4) human subjects only; and (5) evaluation of outcomes related to thoracic surgical procedures or postoperative monitoring and treatment in the context of thoracic oncology. Duplicates were excluded, and reference lists of included studies were manually reviewed to identify additional relevant publications.

Two independent reviewers (PAZ and DEM) conducted data extraction. In the context of this review, failure to rescue was defined as any in-hospital mortality occurring after a postoperative complication in patients undergoing cardiac surgery, which is consistent with the prevailing definitions in the literature.

### 2.2. Data Extraction and Quality Assessment

From each study included in the review, we extracted key data elements, including sample size, patient demographics (age and sex), type of surgical intervention, and reported rates of postoperative complications, mortality, and failure to rescue. We used the Newcastle-Ottawa Scale (NOS) to assess the methodological rigor of non-randomized studies [18]. This tool rates studies on a scale from 0 to 9 stars, with a score of five or above indicating acceptable study quality. Notably, no randomized controlled trials (RCTs) relevant to this topic were identified or included in the analyses. Two reviewers (DEM and AX) independently performed quality assessments, and any disagreements were resolved through discussion to achieve a consensus.

## 3. Results

### 3.1. Search Strategy and Patient Demographics

**Figure 1:**
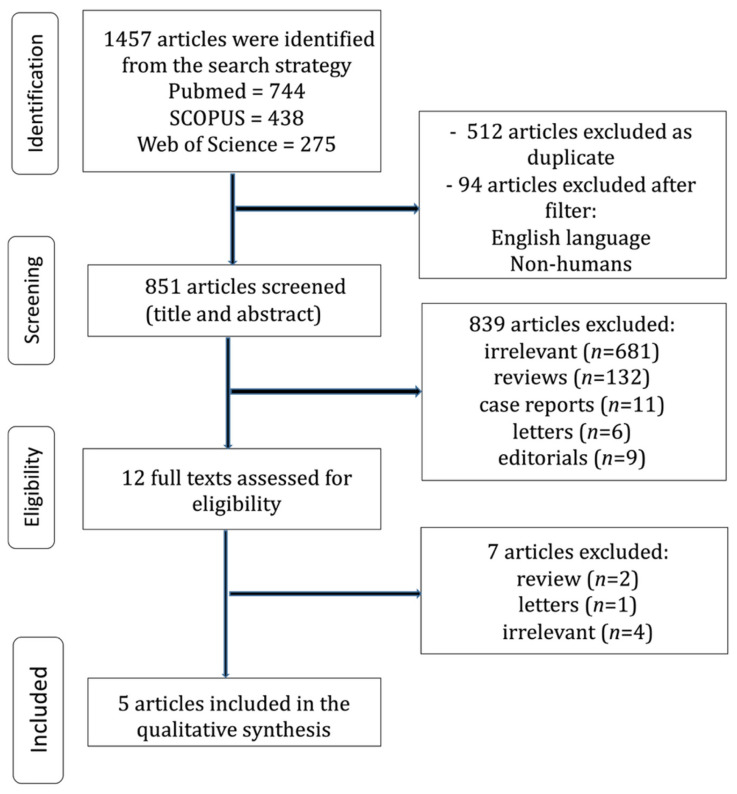
Trial flow of the current review.

**Figure 2:**
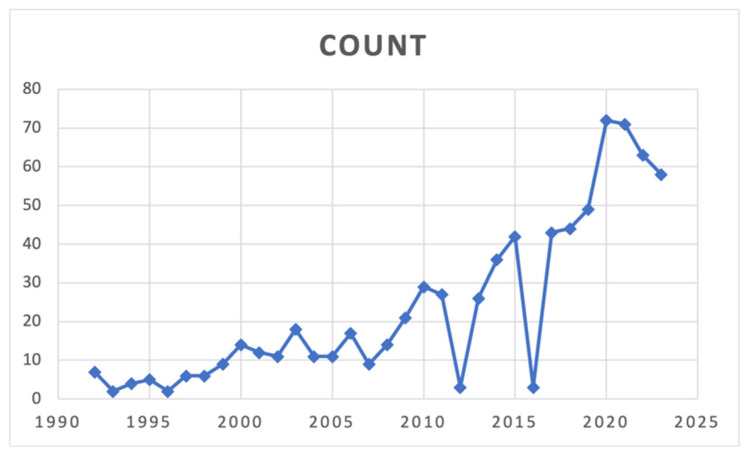
Volume of articles per year in PubMed/Medline on the topic of Failure to Rescue (FTR) in the context of lung resection.

The study selection process is shown in Figure 1. Details of the included studies are summarized in Table 1. A notable upward trend in publications related to rescue (FTR) over the past 30 years is depicted in Figure 2. From an initial pool of 1457 records, five studies [6,19,20,21,22] met all eligibility criteria and were included in the final analysis. The inter-rater reliability between reviewers demonstrated near-perfect agreement, with a kappa coefficient of 0.832 (95% CI: 0.604–1.000). All selected studies were retrospective in design and used data from large, nationally representative databases. These publications spanned from 2015 to 2023. Across the included literature, reported FTR rates varied from 1.7% to 25.9%. Collectively, the studies provided data on 232,678 patients who underwent lung cancer surgery in the United States, Spain, and France. Only five studies met the inclusion criteria, reflecting both the novelty of failure-to-rescue as a research focus in thoracic oncology and the strict eligibility criteria of this review. This highlights the relative scarcity of dedicated studies in this field.

### 3.2. Historical Evolution of the FTR Definition

While the foundational concept of failure to rescue (FTR) hinges on the occurrence of mortality following major complications, its evolution and application in thoracic surgery, particularly in lung cancer resections, have not mirrored the trajectory seen in cardiac surgery, where the definition and scope of FTR have significantly expanded and matured [19,20,21,22,23,24]. A central challenge remains the variability in complication definitions used across institutions, which directly influences reported FTR rates. Institutions that apply broader definitions of major complications tend to report different FTR rates than those using narrower criteria. For example, Bernard et al. [19] characterized FTR as mortality among patients experiencing at least one major complication, including tracheostomy, reintubation, adult respiratory distress syndrome (ARDS), bronchopleural fistula, empyema, pulmonary embolism, pneumonia, reoperation for bleeding, myocardial infarction, stroke, and heart failure. Farjah et al. [20] employed a similar definition. Conversely, Gómez-Hernández et al. [21] utilized the Clavien-Dindo classification system [24] to categorize postoperative complications, which offers a standardized framework for assessing surgical outcomes. Wang et al. [22] adopted a comparable strategy, incorporating a similar number of complication types.

Despite the advantages of the Clavien-Dindo classification in standardizing outcome reporting, it may not be ideally tailored to thoracic oncology patients due to its broad inclusion criteria. To improve consistency in defining FTR across thoracic surgery programs, a Delphi consensus among experienced thoracic surgeons could help establish a refined list of complications most relevant to this patient population. Alternative FTR definitions have also emerged. One approach has suggested focusing on complications that are typically identified early by nursing staff [25]. This nurse-led model included high-risk complications, such as pneumonia, sepsis, gastrointestinal bleeding, deep vein thrombosis, shock, and cardiac arrest. Later, the Agency for Healthcare Research and Quality added acute kidney injury to this list [26]. However, these models have been criticized for narrowing the spectrum of qualifying events and potentially underrepresenting clinically relevant cases, thereby limiting their utility as reliable quality indicators [27].

The development of large-scale clinical registries has significantly contributed to the refinement of FTR definitions. Databases like the American College of Surgeons–National Surgical Quality Improvement Program (ACS-NSQIP) and the Society of Thoracic Surgeons General Thoracic Surgery Database (STS-GTSD) [28,29] offer a high degree of granularity and enable robust risk adjustment. Studies leveraging these data sources allow for more accurate identification of complications and support meaningful comparisons of quality across institutions.

Cardiac surgery has been a valuable model in this context. For instance, Kurlansky et al. [30] validated an institutional performance metric in which FTR was defined as death following one of four specific complications: stroke, renal failure (based on creatinine increase or dialysis requirement), reoperation, or prolonged ventilation (>24 h). The simplicity of this four-event framework facilitates standardization and institutional benchmarking.

Nevertheless, refinement is still needed. Ideally, FTR definitions should emphasize complications that are both potentially fatal and amenable to effective interventions, such as arrhythmias, thromboembolic events, cardiac or respiratory arrest, gastrointestinal perforation, and bleeding or tamponade. Strobel et al. [27] advocated for the inclusion of cardiac arrest in FTR definitions. Meanwhile, the role of reoperation as an FTR component remains controversial. As reintervention often serves as a life-saving measure rather than a failure point, including it as a trigger for FTR may be conceptually flawed. Ultimately, standardizing FTR definitions within thoracic surgery, possibly through a Delphi-based expert consensus, will be essential for ensuring uniform benchmarking, improving quality improvement initiatives, and enabling fair comparisons of surgical outcomes.

### 3.3. Factors Affecting FTR

An in-depth understanding of the different factors that affect and contribute to the success or failure to rescue a patient presenting with a complication following a cardiac surgical operation represents perhaps the first and most crucial step in implementing FTR in clinical practice (Table 2). In the following paragraphs, we attempt to outline the most important aspects in a stepwise manner, starting from the individual patient and reaching the institution-level parameters.

#### 3.3.1. Patient-Level Factors

Clinical status and frailty level contribute to higher postoperative morbidity, mortality, and FTR rates. According to a study [28] that incorporated data from over a million cardiac surgery patients, frailty contributed to a higher FTR rate than non-frail patients. In fact, frail patients were older and had a higher incidence of heart failure and chronic lung, liver, and renal diseases [28]. Although its purpose is not to assess frailty status, the STS risk stratification tool [1] can highlight high-risk patient groups, thus providing an opportunity for increased awareness of their postoperative care needs.

#### 3.3.2. Surgical Complexity

The type and extent of surgical resection directly influence FTR after lung cancer surgery. Among the included studies, Bernard et al. [19] and Gómez-Hernández et al. [21] identified pneumonectomy as one of the strongest independent predictors of rescue failure, reflecting the high perioperative risk profile. In Bernard’s nationwide cohort of 157,566 patients, the national decline in FTR rates from 12.2% in 2005 to 7.1% in 2020 was attributed, in part, to a reduced frequency of pneumonectomies, which decreased from 18.1% in 2005 to 4.8% in 2020. Similarly, Gómez-Hernández and colleagues demonstrated that pneumonectomy doubled the odds of FTR (OR 2.53, 95% CI: 1.07–6.03; *p* = 0.036), underscoring its impact on postoperative rescue.

Minimally invasive approaches appear to mitigate the risk of FTR by reducing the severity of postoperative complications. Bernard et al. [19] reported that video-assisted thoracic surgery (VATS) was associated with both lower complication rates and improved rescue rates compared to open surgery. This observation suggests that surgical techniques not only influence the incidence of complications but also shape the institution’s ability to successfully intervene once complications occur.

Finally, reoperation plays a paradoxical role in the context of rescue. Wang et al. [22] found that reoperation after a major complication was associated with a reduced risk of FTR (OR 0.58, 95% CI: 0.34–0.97; *p* = 0.04), likely reflecting the benefits of timely escalation and proactive surgical intervention. Taken together, these findings underscore that surgical complexity both directly and indirectly modulates the probability of rescue success.

#### 3.3.3. System-Level Challenges

One of the primary institutional determinants influencing FTR rates is whether a healthcare facility functions in an academic or teaching capacity. Grenda et al. [6], analyzing data from the National Cancer Database, demonstrated that hospitals in the highest FTR quartile did not differ in complication rates compared to those in the lowest quartile (36.6% vs. 42.1%, *p* = 0.07), but had significantly higher mortality following complications (9.1% vs. 2.4%, *p* < 0.001). This highlights that the timeliness of recognition and escalation, rather than complication incidence, explains the survival gap. Teaching hospitals are often characterized by the integration of trainees into patient care. However, the impact of training on clinical outcomes and FTR rates remains unclear. Some studies have indicated that the involvement of residents and fellows may enhance perioperative care and improve patient outcomes by ensuring more continuous monitoring and involvement in care processes [29,30]. Nevertheless, these benefits are contingent on the training and competency of the trainees. Adequate education enables prompt recognition of complications and initiation of appropriate escalation protocols. Conversely, insufficient experience or poor supervision may result in delayed recognition and response, thereby increasing the risk of FTR events [2]. These concerns have prompted many centers to incorporate dedicated intensivist consultations, particularly for high-risk or complex postoperative patients [31]. Evidence supports that intensivists often outperform trainees in the critical care management of patients who underwent cardiovascular surgery, with better outcomes in managing postoperative complications [32,33].

Another institutional variable associated with FTR is the surgical volume of the hospital. A growing body of literature supports the observation that high-volume centers tend to have lower FTR rates [34]. This association is thought to stem from the presence of well-established, standardized clinical pathways and institutional familiarity with managing complications in such settings [34]. Notably, analyses of Medicare beneficiaries undergoing major cardiac surgery have revealed comparable complication rates between low- and high-volume institutions, but significantly higher FTR rates in lower-volume settings [35,36]. These findings suggest that the difference lies not in the frequency of complications but in the ability of institutions to intervene effectively once complications occur.

In addition to the physician workforce, staffing levels and qualifications of nurses and allied health professionals significantly influence a hospital’s rescue capacity. Two core dimensions define the quality of hospital staffing: the numerical staffing ratios (e.g., nurse-to-patient or physician-to-patient ratios) and the clinical expertise of personnel. Both factors are associated with improved patient monitoring and reduced FTR rates [2]. Several studies have reported that higher nurse staffing levels are inversely related to the FTR incidence [2,31,32]. When staffing ratios are suboptimal, clinicians may be responsible for larger patient loads, limiting the time and attention that they can devote to each individual. This scenario increases the risk of missing early signs of deterioration, delaying escalation, and ultimately leading to poor outcomes. Additionally, overburdened staff are more prone to communication errors, reduced job satisfaction, and burnout, all of which further compromise patient safety [32]. Hospitals must also pay close attention to off-hour coverage.

#### 3.3.4. Institutional Readiness

At the highest level, institutional characteristics frame the overall capacity to deliver successful rescues. High surgical volume consistently correlates with lower FTR rates across thoracic oncology, as demonstrated in both Bernard’s national study [19] and the Spanish prospective database of Gómez-Hernández et al. [21]. Gómez-Hernández et al. [21] reported that hospitals performing fewer than 120 resections annually had more than twice the odds of rescue failure (OR 2.53, 95% CI: 1.26–5.07; *p* = 0.009). While complication rates were similar between low- and high-volume centers, low-volume institutions exhibited disproportionately higher FTR, suggesting that organizational experience, infrastructure, and standardization of rescue protocols are decisive.

Farjah et al. [20] also observed volume-dependent variations in FTR, reinforcing the principle that institutional readiness, rather than complication incidence, explains survival differences. Academic affiliation, availability of specialized services, and culture of safety further distinguish high-performing centers. Institutions that encourage multidisciplinary collaboration and empower staff to escalate concerns demonstrate superior rescue capacity compared to more hierarchical or fragmented environments.

Institutional readiness also serves as a conceptual bridge to change management frameworks. Reducing FTR requires not only technical expertise but also institutional transformation, aligning leadership priorities, frontline practices, and organizational culture. In this regard, Kotter’s model [16] (as will be described later in the text) provides a structured pathway for embedding rescue into the institutional fabric, ensuring that improvements extend beyond isolated initiatives and become sustainable elements of clinical culture.

### 3.4. Preventive Measures to Minimize Failure-to-Rescue in Lung Cancer Care

Addressing failure-to-rescue in lung cancer surgery requires a multifaceted approach. Implementation of standardized protocols for postoperative care, improvement of communication and coordination among healthcare providers, and investment in ongoing training and education for medical staff are crucial steps. Additionally, leveraging technology to enhance early detection of complications and ensuring adequate staffing and resources in intensive care units are essential for reducing failure-to-rescue incidents. The following preventative strategies can significantly enhance the quality of postoperative care:

1. Enhanced Care Coordination

Effective interdisciplinary collaboration is vital for the timely recognition and management of postoperative complications. Structured communication tools, standardized handoff protocols, and regular multidisciplinary meetings can streamline decision-making and expedite interventions when complications occur [37].

2. Advanced Staff Training and Education

Ongoing training programs ensure that healthcare providers remain proficient in identifying early warning signs, managing postoperative complications, and utilizing emerging technologies. A well-prepared clinical team is better equipped to respond effectively to critical situations, reducing the likelihood of FTR [38].

3. Integration of Advanced Monitoring Technologies

The use of real-time monitoring systems, such as telemetry, wearable sensors, and automated vital sign alerts, enables continuous surveillance of patients after surgery. These tools facilitate early detection of clinical deterioration, allowing for rapid intervention before complications progress [38].

4. Implementation of Standardized Rapid Response Protocols

Therefore, developing clear, institution-wide guidelines for the escalation of care is essential. Protocols that define when and how to activate rapid response teams ensure that critical interventions are delivered promptly and efficiently, thereby improving patient rescue outcomes [39].

5. Patient and Family Engagement

Educating patients and families about potential complications, expected recovery trajectories, and signs that warrant immediate medical attention promotes active participation in their care. Informed and engaged patients are more likely to report symptoms early, supporting faster recognition and treatment of complications [39].

6. Continuous Quality Improvement

Establishing a culture of continuous quality improvement supports the ongoing evaluation of clinical outcomes, complication trends, and response performance. Regular audits, morbidity and mortality reviews, and feedback mechanisms help identify areas for improvement and inform data-driven changes in practices.

By proactively implementing these preventative strategies, healthcare providers can work towards minimizing the occurrence of failure-to-rescue events and optimizing the overall care experience for patients with lung cancer undergoing surgery. This comprehensive approach aims to enhance patient safety, reduce adverse outcomes, and ultimately improve the long-term prognosis and quality of life of individuals affected by lung cancer. While the strategies outlined above are crucial for reducing failure-to-rescue incidents, it is important to address the potential barriers to their successful implementation. Resistance to change, limited resources, and organizational culture are common obstacles that may impede the adoption of standardized protocols and improve communication and coordination among healthcare providers.

### 3.5. Impact of Failure-to-Rescue on Lung Cancer Patients

Failure-to-rescue is a critical determinant of outcomes in lung cancer surgery. Its impact extends far beyond immediate clinical deterioration, profoundly affecting patient prognosis, functional recovery, and psychological well-being. Understanding the downstream effects of FTR underscores the importance of timely interventions, system-level responsiveness, and comprehensive postoperative care.

1. Increased Morbidity and Mortality

FTR is strongly associated with elevated mortality rates and increased postoperative morbidity. When complications such as pneumonia, bleeding, or respiratory failure are not promptly recognized and effectively managed, they can rapidly escalate and jeopardize patient survival. Several studies have shown that hospitals with lower FTR rates, despite having similar complication rates, demonstrate significantly better overall surgical outcomes [40].

2. Prolonged Hospitalization and Delayed Recovery

Patients who experience FTR events often require extended hospital stays and more intensive interventions, such as reoperation or mechanical ventilation. This not only increases healthcare costs but also delays rehabilitation and return to baseline functional status. Moreover, the compounded stress of prolonged hospitalization may increase the risk of secondary complications, such as delirium, hospital-acquired infections, or muscle deconditioning [41].

3. Reduced Quality of Life

Failure-to-rescue has a lasting impact on the quality of life of patients. Unresolved or poorly managed complications can result in chronic pain, fatigue, dyspnea, and decreased mobility, which severely limit the ability to perform daily activities [42]. Patients may also experience long-term dependence on caregivers, loss of independence, and reduced social engagement, all of which diminish their postoperative satisfaction and long-term well-being.

4. Psychological and Emotional Distress

The emotional toll of FTR is significant. Patients who endure unaddressed complications often suffer from increased anxiety, depression, and post-traumatic stress, particularly when recovery is uncertain or traumatic [43]. Family members may also experience emotional exhaustion, caregiver burden, and anticipatory grief. These psychosocial effects can persist well beyond hospital discharge and affect the entire cancer survivorship experience.

The wide-ranging consequences of failure-to-rescue highlight the urgency of strengthening early warning systems, improving team communication, and optimizing resource availability in the provision of surgical care. FTR is not merely a patient-level outcome. Instead, it reflects system-level responsiveness and organizational vigilance. By implementing real-time monitoring technologies, multidisciplinary response protocols, and quality assurance mechanisms, healthcare systems can substantially reduce the incidence of FTR and its adverse impacts. In summary, failure to rescue represents a pivotal challenge in the postoperative management of patients with lung cancer. Its influence on survival, recovery, and quality of life reinforces the need for proactive surveillance and rapid clinical escalation. Healthcare teams must remain committed to early recognition, rapid intervention, and continuous quality improvement to safeguard patients from the profound consequences of FTR.

### 3.6. A Change-Driven Rescue Framework: An 8-Step Roadmap for Reducing Failure-to-Rescue After Lung Cancer Surgery

**Figure 3:**
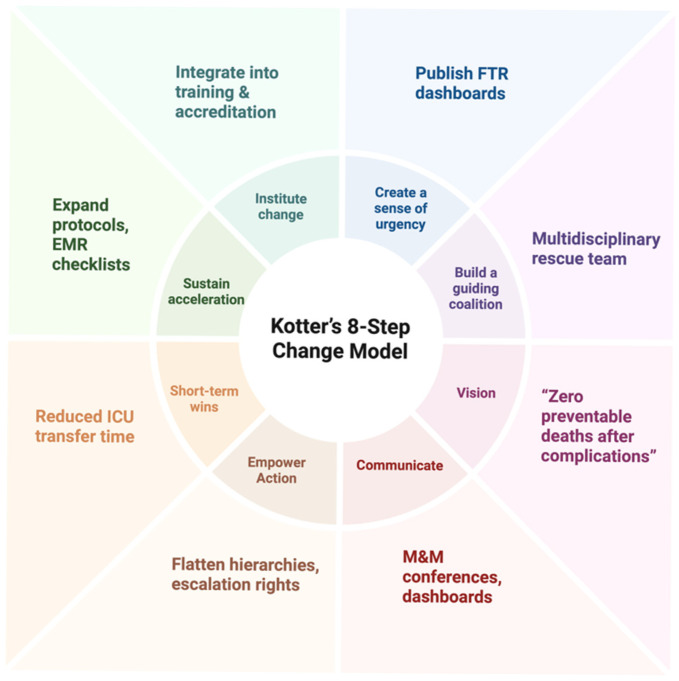
Application of Kotter’s 8-Step Change Model to Improve Failure-to-Rescue Outcomes in Lung Cancer Surgery. Created in BioRender. Magouliotis, D. (2025) https://BioRender.com/6ucturc, accessed on 31 July 2025.

While several frameworks have focused on the clinical aspects of managing postoperative complications, few have integrated institutional transformation strategies that foster a long-lasting reduction in FTR events. Having illustrated the multifactorial determinants of FTR, Kotter’s model [16] provides a cohesive framework for translating these insights into organizational transformation. Each of Kotter’s steps aligns with practical strategies in thoracic oncology, from creating urgency with FTR dashboards to consolidating gains through standardized rescue protocols (Figure 3). This model recognizes that timely rescue from complications is not only a clinical act but also a systemic behavior that requires leadership engagement, empowered teams, and adaptive learning.

1. Establish a Sense of Urgency: Recognize FTR as a Quality Crisis

Before any clinical pathway is implemented, institutions must treat high FTR rates as a critical failure of the system, and not just the bedside team. By transparently reporting unit-level FTR events and benchmarking them against peers, leaders create a sense of urgency for change. Real cases in which complications escalated due to delays should be shared to highlight the cost of inaction.

Example metric: Demonstrating that the FTR in pneumonectomy patients exceeds the national average emphasizes the need for immediate action.

2. Build a Guiding Coalition: Form a Multidisciplinary Rescue Team

Establish a dedicated “Rescue Task Force” composed of thoracic surgeons, anesthesiologists, critical care physicians, advanced practice nurses, respiratory therapists, and quality improvement experts. This team should be responsible for developing, deploying, and monitoring the rescue strategy.

Example action: A multidisciplinary rescue team, including thoracic surgeons, intensivists, anesthesiologists, nurses, and rapid response coordinators, can serve as the core group driving change.

3. Develop a Unified Vision and Protocolized Rescue Pathway

The coalition’s mission is translated into a protocolized pathway that includes (1) early warning criteria for respiratory and hemodynamic deterioration, (2) decision trees for escalation of care, and (3) rescue timelines (e.g., ICU transfer within 30 min of trigger).

In contrast to standard enhanced recovery after surgery (ERAS), this pathway is reactive and is designed specifically for post-complication salvage.

Example tool: Defining a vision, such as “zero preventable deaths after complications,” and initiatives like standardized escalation protocols or mandatory early warning scores create a shared institutional goal.

4. Communicate the Vision for Buy-In

Regular departmental meetings, surgical morbidity and mortality (M&M) conferences, and internal dashboards can reinforce the importance of FTR reduction. Using storytelling (e.g., anonymized case narratives) can help personalize the vision for the staff.

Example tool: Visual “Rescue Flowchart” above nurse stations, summarizing steps in case of sudden desaturation or hypotension.

5. Empower Others to Act on the Vision: Break Down Barriers

Removing barriers, such as hierarchical communication delays, allows junior staff to escalate concerns without fear of reprisal. Empowering nurses and residents with the authority to activate rapid response systems is a critical step.

Example policy: Empower nurses to activate a “Rescue Alert” protocol without requiring initial physician’s permission.

6. Generate Short-Term Wins: Track and Celebrate Early Successes

Early measurable successes may include reduced median recognition-to-ICU transfer time, lower pneumonia-related FTR within six months, or increased compliance with escalation protocols.

Example metric: “Rescue Without ICU” ratio, measuring patients rescued successfully on the ward.

7. Consolidate Gains and Produce More Change: Refine and Expand

Building on early improvements, institutions can expand protocols to cover additional complications (e.g., acute coronary events and pulmonary embolism), integrate checklists into electronic health records, and broaden training initiatives.

Example adaptation: Add lung function thresholds (e.g., ppoFEV1 <40%) as triggers for enhanced surveillance.

8. Anchor New Approaches in the Culture: Make Rescue a Shared Value

Long-term sustainability requires embedding FTR reduction into hospital quality indicators, residency curricula, and accreditation standards. Over time, successful rescue must be perceived as a defining marker of institutional excellence.

Example initiative: Annual “Rescue Excellence Award” to recognize frontline staff who made timely life-saving interventions.

Despite its conceptual appeal, the application of Kotter’s framework in surgical settings is not without its barriers. One of the most pervasive challenges is the hierarchical nature of surgical culture, where junior staff may hesitate to escalate concerns or activate rapid response pathways in the presence of senior surgeons. This dynamic undermines Kotter’s emphasis on empowering broad-based actions.

Fragmented communication between departments is another obstacle. Effective rescue often requires coordination across thoracic surgery, anesthesiology, intensive care units, and nursing units. Misaligned priorities, unclear escalation responsibilities, and siloed workflows can delay recognition and intervention, directly increasing FTR.

Furthermore, institutional inertia and resource limitations may hinder the creation of a sense of urgency or consolidation of short-term wins. Hospitals with constrained ICU capacity or insufficient staffing may struggle to implement systemic changes, even when motivated. Finally, cultural resistance to change, particularly in high-volume centers with entrenched practices, can limit the anchoring of new approaches into the daily workflow.

Recognizing these barriers is critical because they highlight the need for adaptive strategies tailored to the surgical environment. Successful implementation of Kotter’s model in thoracic oncology, therefore, requires not only leadership commitment but also deliberate efforts to flatten hierarchies, foster interdepartmental communication, and align institutional resources with the shared vision of improving rescue.

### 3.7. Building a Culture of Reflection: Continuous Review and Adaptation in Lung Cancer Surgery Pathways

Even in high-performing thoracic surgical programs, improvement is an ongoing process. In the context of lung cancer resections, where patients are often elderly, frail, or have limited cardiopulmonary reserve—rigid protocols alone are insufficient. Instead, dynamic reflection and continuous learning are essential to ensure responsiveness to evolving challenges.

Data managers hiring. These professionals are devoted to data mining and analysis in collaboration with statisticians. This is the first step in allowing further review of the effectiveness of current practices.

Two core drivers foster this adaptive environment: a structured post-hoc review of clinical outcomes and the integration of emerging oncologic, perioperative, and surgical evidence. To institutionalize this culture of continuous learning, we propose a multi-layered feedback framework grounded in real-time data analysis, enriched by cross-disciplinary insights, and reinforced through collaborative accountability.

(1) Creation of a Thoracic Outcomes Intelligence Unit (TOIU)

Beyond traditional data managers, institutions should develop dedicated units focused exclusively on lung cancer surgical outcomes, including complications, readmissions, and oncologic efficacy (e.g., margin status and lymph node harvest). This unit should integrate thoracic surgeons, data analysts, and oncology nurses to identify trends and develop early warning indicators for quality lapses.

Key insight: An increase in prolonged air leak rates or unexpected ICU transfers may indicate a flaw in the intraoperative technique or postoperative chest tube protocols.

(2) Multidisciplinary Complication Review Boards (MCRBs)

Unlike general morbidity and mortality (M&M) conferences, multidisciplinary complication review boards (MCRBs) should include not only thoracic surgeons but also pulmonologists, oncologists, anesthesiologists, and critical care teams. Their scope extends beyond reviewing deaths to encompass complex rescues and near misses following lung resections. Each case is examined for potential delays in escalation, overlooked radiologic or clinical warning signs, and breakdowns in communication. Emphasis is placed not on attributing blame but on fostering a culture of shared system responsibility.

(3) Failure Pattern Mapping (FPM)

Adapted from root-cause analysis models, this tool identifies recurrent micro-failures within the lung cancer care continuum. For instance, delays in recognizing a bronchopleural fistula, inappropriate fluid management in patients with borderline FEV1, or the omission of early CT imaging in febrile post-lobectomy patients are typical examples. By systematically capturing such events, failure pattern mapping (FPM) transforms anecdotal learning into visualized patterns of care breakdown, which can then guide targeted team training and protocol revision.

(4) Real-Time Benchmarking Dashboards

Institutions should implement dashboards that continuously track outcome metrics, such as the FTR rate after lobectomy or pneumonectomy, time to chest tube removal, and 30-day readmission rate for respiratory complications. Crucially, these dashboards should allow comparison of individual surgeon and team performance against both internal peers and external benchmarks, such as STS or ESTS data, with results presented transparently and unblinded during reviews.

(5) Thoracic Quality Symposia (TQS)

Held biannually, these internal forums provide a platform to present outcome data openly, invite guest discussants to critique institutional practices, and showcase successful ‘rescue stories’ that highlight the factors enabling favorable outcomes. Unlike routine staff meetings, thoracic quality summits (TQS) emphasize a learning culture in which innovation and transparency are recognized and valued over hierarchy.

(6) Incorporation of Evolving Evidence into Practice Loops

The thoracic oncology field is rapidly evolving with the introduction of novel neoadjuvant therapies, lung-sparing techniques, and immunotherapy protocols. Institutions must therefore establish pathways for the rapid assimilation of new evidence into perioperative care. For instance, patients treated with neoadjuvant immunotherapy may develop increased rates of fibrosis or adhesions, necessitating adjustments in surgical strategies and tailored postoperative monitoring protocols. To facilitate this process, the Thoracic Oncology Innovation Unit (TOIU), in collaboration with the tumor board, should issue timely practice briefs whenever new clinical trials or guidelines are published, ensuring that frontline staff remain aligned with emerging standards.

## 4. Discussion

Failure to rescue (FTR), defined as in-hospital mortality following a major postoperative complication, has emerged as a critical metric for evaluating the quality of surgical and perioperative care. While traditional quality indicators have focused primarily on complication rates and mortality, growing evidence supports the notion that a hospital’s ability to effectively manage complications is a more meaningful determinant of patient outcomes, especially in the field of thoracic surgery.

Our review confirms that in patients undergoing lung cancer resection, wide variations exist not only in the incidence of complications but also in FTR rates. Across the included studies, FTR ranged from 1.7% to 25.9%, underscoring significant institutional heterogeneity in postoperative rescue capabilities. Interestingly, these variations were not uniformly correlated with complication rates. Instead, consistent with previous findings by Farjah et al. [19] and Grenda et al. [6], hospitals with similar complication rates often exhibited markedly different FTR rates, implying that hospital-level factors (such as intensive care protocols, nursing ratios, surgical expertise, and early detection systems) play a critical role.

Several institutional and patient-related predictors of FTR have emerged in the literature. High surgical volume, thoracic surgery specialization, and use of minimally invasive approaches (e.g., VATS or robotic surgery) were consistently associated with lower FTR rates. For example, Bernard et al. [19] demonstrated a national decline in FTR rates in France from 12.2% in 2005 to 7.1% in 2020, attributing this improvement, in part, to reduced pneumonectomy rates and increased use of VATS. Similarly, Gómez-Hernández et al. [21] identified low hospital volume and pneumonectomy as independent predictors of rescue failure in a prospective, national cohort. Patient-level characteristics, particularly advanced age, also appear to increase susceptibility to FTR. Wang et al. [22] found that each decade of age increased the odds of FTR by 55%, with elderly patients being less likely to undergo reintervention or rescue measures, further highlighting a potential gap in equity of care. Notably, in this cohort, reoperation was paradoxically associated with lower FTR, likely reflecting timely escalation of care rather than treatment futility [22].

These findings support a shift in quality improvement efforts from merely reducing complications to actively enhancing early recognition and treatment pathways. Institutional strategies may include rapid response teams, postoperative early warning systems, simulation-based staff training, and stricter adherence to enhanced recovery protocols. As previously suggested by Silber et al. [9] and reinforced by contemporary studies, the essence of improving postoperative survival lies in structured and timely intervention rather than solely technical performance. This review represents the first attempt to systematically bridge the evidence of FTR in lung cancer surgery with organizational change frameworks. While existing studies highlight clinical predictors such as pneumonectomy, comorbidities, and hospital volume, their translation into actionable institutional strategies has been limited. By embedding Kotter’s model throughout the discussion, we provide a novel roadmap for aligning patient- and system-level insights with organizational preparedness.

This review has several limitations. First, only five studies met the inclusion criteria, reflecting both the novelty of applying FTR as a quality indicator in thoracic oncology and the strict eligibility thresholds of the search strategy. Therefore, the generalizability of our conclusions is restricted, and the quantitative evidence base remains limited. Second, all the included studies were retrospective analyses that relied largely on administrative or registry data. These datasets lack granularity in important domains such as tumor stage, functional status, and intraoperative variables, which may confound the interpretation of FTR determinants. Moreover, definitions of FTR varied across studies, ranging from Clavien-Dindo classification to database-specific definitions, complicating direct comparisons. Third, while this is the first review to systematically integrate organizational change theory, specifically Kotter’s model, into the analysis of FTR in lung cancer surgery, the practical application of this framework has not yet been validated in thoracic surgical settings. Hierarchical structures, fragmented communication, and resource constraints may limit feasibility; therefore, our recommendations should be viewed as hypothesis-generating rather than prescriptive. Finally, the review was limited to English-language studies and did not include unpublished data, which may have introduced potential publication bias. Future prospective multicenter studies, particularly those evaluating structured rescue protocols and change management interventions, are needed to confirm the findings and test the applicability of Kotter’s model in real-world practice.

In conclusion, FTR offers a nuanced and actionable perspective on perioperative mortality following lung cancer resection. Beyond surgical excellence, institutional readiness to detect and manage complications decisively shapes outcomes. Tailored interventions at both the patient and hospital levels have the potential to markedly reduce mortality and improve the quality of thoracic oncologic care.

## 5. Conclusions

Failure to rescue is a critical and modifiable determinant of surgical outcomes in patients undergoing lung cancer resection. As complication rates alone do not explain variations in postoperative mortality, the ability of institutions to recognize and respond effectively to these complications defines the true quality of care. This review highlights the multifactorial nature of FTR, emphasizing patient-related vulnerabilities, system-level inefficiencies, and cultural barriers to healthcare delivery. To address these challenges, we propose a comprehensive approach that combines clinical vigilance and structural reforms. Early recognition, timely escalation, and effective management must be embedded within a proactive institutional culture supported by real-time data analysis, multidisciplinary collaboration, advanced monitoring technologies, and continuous education. Importantly, we highlight the need for adaptive rescue frameworks that go beyond clinical algorithms and incorporate change management principles, such as Kotter’s 8-step model, to foster sustainable improvement. Ultimately, minimizing FTR is not a matter of protocol adherence alone; it reflects a health system’s readiness, responsiveness, and resilience. By transforming how we monitor, respond to, and learn from postoperative complications, thoracic surgery programs can elevate patient safety, optimize outcomes, and redefine standards of care in lung cancer surgery.

## Figures and Tables

**Table 1:** Baseline characteristics of the studies and patients included in the present review. Abbreviations: FTR = Failure to Rescue; NOS = Newcastle-Ottawa Scale; R = Retrospective; STS = Society of Thoracic Surgeons; NCDB = National Cancer Database; ACS = American College of Surgeons; NSQIP = National Surgical Quality Improvement Program; N/A = Not Available; n = number.

Study ID, Year	Country/Database	Study Design	Definition of FTR	Study Population, n	Adverse Events, %	Mortality, %	FTR, %	Statistical Notes	NOS
Grenda 2015 [6]	USA, NCDB	R	Death after major complication (e.g., pneumonia, ARDS, PE, MI, sepsis)	645	15.6–23.3	1.4–6	8.7–25.9	Logistic regression; *p*-values for hospital-level predictors	7
Bernard 2023 [19]	France, National Database	R	Death after severe cardiopulmonary complication (pneumonia, PE, ARDS)	157,566	27.8	Reduced from 3.8% in 2005 to 2.9% in 2020	Reduced from 12.2% in 2005 to 7.1% in 2020	Multivariable analysis, CI reported	7
Farjah 2015 [20]	USA, STS Database	R	Death after ≥ 1 postoperative major complication (STS defined)	30,000	36–42	0.7–3.2	1.7–6.8	Adjusted OR for hospital volume; CI reported	7
Gómez-Hernández 2023 [21]	Spain, National Database	R	In-hospital mortality after Clavien-Dindo grade ≥ IIIb complication	3533	10.2	1.7	16.3	Kaplan-Meier and logistic regression, *p*-values	7
Wang 2023 [22]	USA, ACS NSQIP	R	Death following ≥ 1 major complication (NSQIP-defined: pneumonia, sepsis, unplanned intubation, etc.)	40,934	10.1	N/A	5.6–12	OR per decade age (1.55, *p* < 0.001), subgroup *p*-values reported	7

**Table 2:** Risk Factors Contributing to Failure-to-Rescue in Lung Cancer Surgery.

Category	Risk Factor	Description
Surgical Complexity	Procedure Type and Extent of Resection	Complex procedures and extensive resections (e.g., pneumonectomy) increase complication risk and reduce the likelihood of successful rescue.
	Patient Comorbidities	Coexisting conditions (e.g., cardiac disease, poor pulmonary reserve) raise the risk of adverse outcomes postoperatively.
Postoperative Monitoring	Inadequate Surveillance	Delayed detection of complications due to insufficient monitoring protocols or clinical vigilance can lead to missed rescue opportunities.
Care Coordination	Poor Multidisciplinary Communication	Lack of effective handoffs or collaboration between surgeons, anesthesiologists, intensivists, and nurses delays appropriate interventions.
Resource Allocation	Staffing Shortages and Inadequate ICU Resources	Limited staff, overwhelmed ICUs, and a lack of essential equipment impair the ability to intervene quickly and appropriately after complications arise.

## Data Availability

The data that support the findings of this study are available from the corresponding author upon reasonable request.
